# Improving the Covid-19 Vaccination Rate in Pakistan—A Multipronged Policy Approach

**DOI:** 10.3389/fpubh.2021.729102

**Published:** 2021-09-03

**Authors:** Muhammad Salar Khan

**Affiliations:** Schar School of Policy and Government, George Mason University, Arlington, VA, United States

**Keywords:** vaccine hesitancy, Covid-19 vaccine, nudge, collaborative approach, legislation, policy approach, Pakistan

In Pakistan, the Covid-19 vaccine hesitancy is a real challenge amid all the rumors and disinformation about the vaccine (1). As a researcher who is witnessing the reckless manner in which Pakistanis are reacting to the vaccine (2), the observation raises red flags and grave concerns for fellow countrymen. To overcome all the drivers of vaccine hesitancy, the government must employ an effective policy strategy informed by nudge behavioral science, appropriate legislation, active community collaboration, and previous learning. Such a multipronged policy approach will increase the vaccine take-up rate in the country.

While Pakistan has supply-side challenges, it still has procured over 40 million Covid-19 vaccine doses from the World Health Organization, China, the US, the UK, and Germany (3–6). Aiming to vaccinate about 70 million people by the end of 2021, the Government of Pakistan recently committed $1.1 billion to procure the Covid-19 vaccine (7). As Pakistan further mitigates its supply-side challenges, it needs to pay heed to the demand-side hurdles as well.

Compared to the first three waves (8, 9), the fourth wave of Covid-19 has been extremely lethal (10), causing a severe strain on the already burdened healthcare system in the country. Hesitancy toward the Covid-19 vaccine, driven by various factors ranging from cultural and religious beliefs (2) and concerns about vaccines' side effects (11) to many half-baked conspiracy theories and social media rumors about the vaccine's reliability, origin, and efficacy, further adds to the strain (1, 2). Consequently, the country has been able to vaccinate only 18.2% of its population, as per the recent statistics shared by the Government of Pakistan (10). If the vaccine hesitancy is not countered, it will take a long period to return to a state of normalcy from the pandemic.

The Government of Pakistan launched the Covid-19 vaccination drive early this year initially for healthcare workers and older people (10). To increase the vaccination rate, the government recently opened the vaccination drive to all adults free of cost (10). In a country where mobile users are over 70% (as per the World Bank's statistics), it extensively used mass communication through mobile phones to encourage people to vaccinate and tackle the pandemic (12). However, the country still needs to adopt more strategic measures to reduce the vaccination hesitancy of locales.

One way to fight Covid-19 vaccination hesitancy is to incorporate a nudge behavioral science approach in the government's strategy to increase the vaccination rate (13). Such an approach calls for “nudges,” which simply are processes, adjustments, or structures meant to guide people toward a particular behavioral choice (14). Such nudges can also include financial or non-financial incentives that can induce people to take the vaccines. For example, the state of Ohio, United States of America, recently enacted a lottery program to boost Covid-19 vaccination rates. According to a story published by *The Washington Post* highlighting the impact of the program, the state recorded a 28 percent increase in the vaccination rate of those aged 16 and older in the program's first week (15). Heterogeneity among regions and people will necessitate a contextualized and personalized nudge approach when dealing with Covid-19 vaccination hesitancy. In Pakistan, the government can also employ its unique nudge approach by awarding cash and non-cash benefits conditional upon people's sign-up for the Covid-19 vaccination.

Cash benefits could be integrated into the existing social safety net programs, for instance, Benazir Income Support Program (BISP) and the Ehsaas Cash Program. The government can encourage those who are availing benefits under such cash programs to earn additional cash if they complete their vaccination certificate. Further, relatively economical non-cash programs can also work. Under one such intervention program implemented in India by the MIT's Abdul Latif Jameel Poverty Action Lab, Noble Laureates Banerjee and Duflo demonstrated that free provision of 1 kg of lentils to people who vaccinated their children led to significant improvement in local healthcare outcomes (16). In Pakistan, similar policies of providing incentives will particularly increase the vaccination rate among less-educated, ill-informed poor people, who are generally more prone to rumors and conspiracy theories than the educated upper and middle classes.

Similarly, by employing the nudge approach, the Government of Pakistan should further strengthen its existing mass communication strategy by including slogans that appeal to people's self-interest and altruism. For instance, instead of “vaccinate for your life,” a revised slogan could be “vaccinate for your life and your loved ones' life.”

In addition, this nudge approach should be complemented with proper legislation mandating workplaces to give breaks to their employees for completing the vaccination period. Likewise, the city government units should approach the administration of colleges and universities, ensuring that students show vaccination proof to be allowed entry to the premises of their institute.

Lastly, the government should involve other vital stakeholders, such as non-governmental organizations, health professionals, community leaders, and *ulemas* (religious scholars), to spread awareness about vaccination. Particularly, in a country where ulemas are revered and looked up to guidance, they should be engaged in squashing all the rumors around the vaccine and spreading the importance of disease prevention. They should mention the narration in which the Prophet Muhammad (PBUH) advised to prevent the spread of contagious diseases. Thus, they should remind and educate the public that prevention in the form of vaccination is obligatory for all.

Pakistan, which has a track record of slow immunization for other lethal diseases such as HBV and Polio, must also revive its memory of how it increased immunization in the past. Indeed, a collaborative approach implemented with the help of international partners and other interventions were introduced to amplify immunization rates (17). The Government of Pakistan can revisit its history. For example, the government can check who has not received HBV or Polio vaccine in the past. This information may also inform who might be hesitant to the Covid-19 vaccine.

Alongside the government's introspection into its institutional and procedural memory of combating low vaccination rates for previous non-Covid diseases, it is proposed to implement the multipronged policy approach outlined here (and summarized in Figure 1) to reduce the prevailing Covid-19 vaccination hesitancy of locales. It is time to act now!

**Figure 1:**
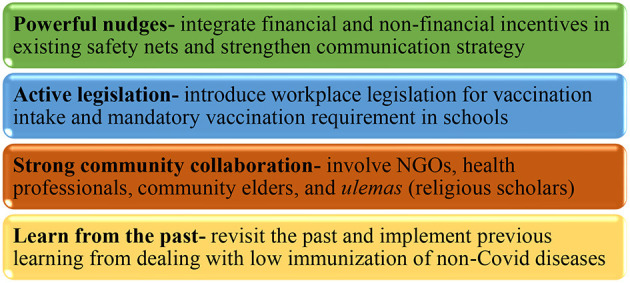
A coherent, multipronged policy strategy for dealing with Covid-19 vaccination hesitancy in a low-middle-income country, Pakistan. The four suggestions presented in this commentary work together to increase vaccination rates in Pakistan.

## Author Contributions

MK conceptualized, analyzed, and wrote the manuscript.

## Conflict of Interest

The author declares that the research was conducted in the absence of any commercial or financial relationships that could be construed as a potential conflict of interest.

## Publisher's Note

All claims expressed in this article are solely those of the authors and do not necessarily represent those of their affiliated organizations, or those of the publisher, the editors and the reviewers. Any product that may be evaluated in this article, or claim that may be made by its manufacturer, is not guaranteed or endorsed by the publisher.
